# QT interval and short-term outcome in acute heart failure

**DOI:** 10.1007/s00392-023-02173-9

**Published:** 2023-04-01

**Authors:** Òscar Miró, Oriol Aguiló, Joan Carles Trullàs, Víctor Gil, Begoña Espinosa, Javier Jacob, Pablo Herrero-Puente, Josep Tost, María Luísa López-Grima, Pere Comas, Carlos Bibiano, Lluís Llauger, Enrique Martin Mojarro, María Pilar López-Díez, Julio Núñez, Zubaid Rafique, Kelly R. Keene, Frank Peacock, Pedro Lopez-Ayala, Christian Mueller, Manuel Montero Pérez-Barquero, Lluís Mont, Pere Llorens, the ICA-SEMES research investigators, Marta Fuentes, Cristina Gil, Héctor Alonso, Enrique Pérez-Llantada, Francisco Javier Martín-Sánchez, Guillermo Llopis García, Mar Suárez Cadenas, Rosa Escoda, Sira Aguiló, Carolina Sánchez, Javier Millán, José Pavón, Antonio Noval, María Luisa López-Grima, Amparo Valero, María Ángeles Juan, Alfons Aguirre, Maria Àngels Pedragosa, Silvia Mínguez Masó, María Isabel Alonso, Francisco Ruiz, José Miguel Franco, Ana Belén Mecina, Marta Berenguer, Ruxandra Donea, Susana Sánchez Ramón, Virginia Carbajosa Rodríguez, Pascual Piñera, José Andrés Sánchez Nicolás, Raquel Torres Garate, Aitor Alquézar-Arbé, Miguel Alberto Rizzi, Sergio Herrera, Alex Roset, Irene Cabello, Antonio Haro, Fernando Richard, José María Álvarez Pérez, Pablo Herrero Puente, Joaquín Vázquez Álvarez, Belén Prieto García, María García García, Marta Sánchez González, Patricia Javaloyes, Inmaculada Jiménez, Néstor Hernández, Adriana Gil, Francisca Molina, Tamara García, Juan Antonio Andueza, Rodolfo Romero, Martín Ruíz, Roberto Calvache, María Teresa Lorca Serralta, Luis Ernesto Calderón Jave, Beatriz Amores Arriaga, Beatriz Sierra Bergua, Enrique Martín Mojarro, Brigitte Silvana Alarcón Jiménez, Lisette Travería Bécquer, Guillermo Burillo, Lluís Llauger García, Gerard Corominas LaSalle, Carmen Agüera Urbano, Ana Belén García Soto, Elisa Delgado Padial, Ester Soy Ferrer, María Adroher Múñoz, José Manuel Garrido, Francisco Javier Lucas-Imbernón, Rut Gaya, Carlos Bibiano, María Mir, Beatriz Rodríguez, José Luis Carballo, Esther Rodríguez-Adrada, Belén Rodríguez Miranda, Monika Vicente Martín, Pere Coma Casanova, Joan Espinach Alvarós

**Affiliations:** 1 Emergency Department, Hospital Clínic, Institut d’Investigació Biomèdica August Pi i Sunyer (IDIBAPS), University of Barcelona, Villarroel 170, 08036 Barcelona, Catalonia Spain; 2 The GREAT (Global Research on Acute Conditions Team) Network, Rome, Italy; 3 Laboratori de Reparació i Regeneració Tissular (TR2Lab), Emergency Department, Hospital d’Olot, Girona, Medical School, Universitat de Vic-Central de Catalunya, Barcelona, Catalonia Spain; 4 Laboratori de Reparació i Regeneració Tissular (TR2Lab), Internal Medicine Department, Hospital d’Olot, Girona, Medical School, Universitat de Vic-Central de Catalunya, Barcelona, Catalonia Spain; 5 Emergency Department, Short-Stay Unit and Home Hospitalization, Hospital Doctor Balmis, Alicante, Spain; 6 Emergency Department, Hospital Universitari de Bellvitge, L’Hospitalet de Llobregat, Barcelona, Catalonia Spain; 7 Emergency Department, Hospital Universitario Central de Asturias, Oviedo, Spain; 8 Emergency Department, Hospital de Terrassa, Barcelona, Catalonia Spain; 9 Emergency Department, Hospital Doctor Peset, Valencia, Spain; 10 Emergency Department, Hospital Sant Joan de Déu de Martorell, Barcelona, Catalonia Spain; 11 Emergency Department, Hospital Infanta Leonor, Madrid, Spain; 12 Emergency Department, Hospital Universitari de Vic, Barcelona, Catalonia Spain; 13 Emergency Department, Hospital Sant Pau i Santa Tecla, Tarragona, Catalonia Spain; 14 Emergency Department, Hospital Universitario de Burgos, Burgos, Spain; 15 Cardiology Department, Hospital Clínico de Valencia, INCLIVA, Valencia, Spain; 16 Emergency Department, Ben Taub Hospital, Baylor College of Medicine, Houston, TX USA; 17 Cardiovascular Research Institute Basel (CRIB) and Cardiology Department, University Hospital Basel, Basel, Switzerland; 18 Internal Medicine Department. Hospital Universitario Reina Sofía, IMIBIC, Universidad de Córdoba, Córdoba, Spain; 19 Arrhythmia Section, Cardiology Department, Hospital Clínic, Institut d’Investigació Biomèdica August Pi i Sunyer (IDIBAPS), University of Barcelona, Barcelona, Catalonia Spain

**Keywords:** Acute heart failure, Electrocardiogram, QTc interval, Outcome, Mortality, Emergency department

## Abstract

**Objective:**

To investigate the association of corrected QT (QTc) interval duration and short-term outcomes in patients with acute heart failure (AHF).

**Methods:**

We analyzed AHF patients enrolled in 11 Spanish emergency departments (ED) for whom an ECG with QTc measurement was available. Patients with pace-maker rhythm were excluded. Primary outcome was 30-day all-cause mortality and secondary outcomes were need of hospitalization, in-hospital mortality and prolonged hospitalization (> 7 days). Association between QTc and outcomes was explored by restricted cubic spline (RCS) curves. Results were expressed as odds ratios (OR) and 95%CI adjusted by patients baseline and decompensation characteristics, using a QTc = 450 ms as reference.

**Results:**

Of 1800 patients meeting entry criteria (median age 84 years (IQR = 77–89), 56% female), their median QTc was 453 ms (IQR = 422–483). The 30-day mortality was 9.7%, while need of hospitalization, in-hospital mortality and prolonged hospitalization were 77.8%, 9.0% and 50.0%, respectively. RCS curves found longer QTc was associated with 30-day mortality if > 561 ms, OR = 1.86 (1.00–3.45), and increased up to OR = 10.5 (2.25–49.1), for QTc = 674 ms. A similar pattern was observed for in-hospital mortality; OR = 2.64 (1.04–6.69), for QTc = 588 ms, and increasing up to OR = 8.02 (1.30–49.3), for QTc = 674 ms. Conversely, the need of hospitalization had a U-shaped relationship: being increased in patients with shorter QTc [OR = 1.45 (1.00–2.09) for QTc = 381 ms, OR = 5.88 (1.25–27.6) for the shortest QTc of 200 ms], and also increasing for prolonged QTc [OR = 1.06 (1.00–1.13), for QTc = 459 ms, and reaching OR = 2.15 (1.00–4.62) for QTc = 588 ms]. QTc was not associated with prolonged hospitalization.

**Conclusion:**

In ED AHF patients, initial QTc provides independent short-term prognostic information, with increasing QTc associated with increasing mortality, while both, shortened and prolonged QTc are associated with need of hospitalization.

**Graphical abstract:**

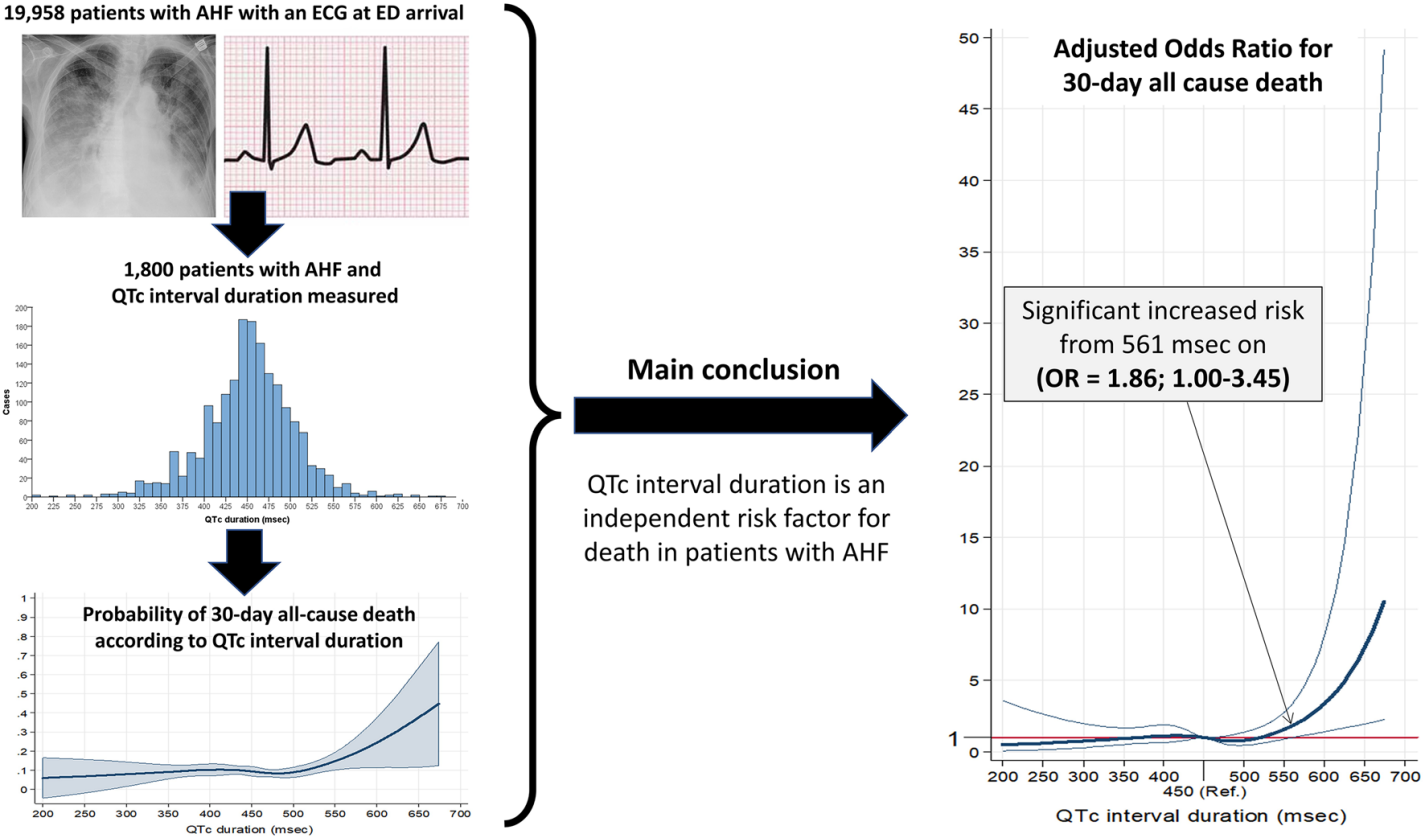

**Supplementary Information:**

The online version contains supplementary material available at 10.1007/s00392-023-02173-9.

## Introduction

Heart failure (HF) is highly prevalent in people over the age of 65 years of age, and constitutes a leading cause of hospitalization and death [1, 2]. Decompensations, termed acute HF (AHF), are a crucial point in the natural history of HF, because most deaths and health care costs are associated with this presentation [2, 3]**.** Therefore, it is a priority to identify risk factors linked to AHF and adverse outcomes, especially death, to help us to understand and risk stratify patients. This may result in areas of intervention or future research to improve prognosis of patients with HF [4, 5].

A 12-lead electrocardiogram (ECG) is recommended by European Society of Cardiology (ESC) guidelines to be performed early in the evaluation of patients presenting with AHF, as it allows prompt identification of decompensation triggers, especially coronary ischemia and abnormal rhythms such as rapid atrial fibrillation [6]. Alternatively, less attention has been paid to other components of the ECG in patients with AHF. Among them, the QT interval could provide useful information to emergency physicians when evaluating the severity of AHF and the risk of developing short-term adverse events. It is important to note that, as the QT interval reflects the duration of ventricular depolarization and repolarization, its duration is a function of heart rate. Thus, a heart rate correction (the QT corrected; QTc) is necessary to standardize its measurement [7]. There are four different commonly used methods to calculate QTc; two exponential (Bazett and Fridericia methods) [8, 9] and two linear (Framingham et al. and Hodges et al. methods) [10, 11]. When corrected, the QTc is equal the QT at a heart rate of 60 beats per minute.

Because of the electrical instability and arrhythmogenesis associated with prolonged QTc, QTc duration has been considered useful as prognosticator of long-term adverse outcomes. Using the QTc, several authors have reported a J-shaped association between QTc duration and some adverse events in healthy people and patients with cardiovascular risk factors, including HF [12–18]. However, less evidence exists about the potential utility of QTc duration as marker of decompensation severity and short-term risk for adverse events in patients with AHF. The association between QTc duration and long-term survival was analyzed in a Swiss cohort of 173 consecutive ED AHF patients that had been included in the B‐Type Natriuretic Peptide for Acute Shortness of Breath Evaluation (BASEL) study during 2001–2002 and followed during 2 years [19]. Although QRS duration was found related to mortality, the authors failed to demonstrate a relationship between QTc and death risk. This lack of correlation may have been a function of the fact that QTc duration was handled as a binary variable, defined as abnormal if > 440 ms. A more recent study using the Korean AHF (KorAHF) registry analyzed 4990 hospitalized patients during 2011–2014 and followed a median 44 months. They used restricted cubic spline (RCS) curves, to allow a more dynamic analysis of the relationship between QTc and long-term mortality, and reported a J-shaped relationship between QTc and mortality for both sexes, with a higher risk of dying for males [20]. Therefore, a more complete understanding of association between QTc and short-term outcomes in patients with AHF, not only limited to death risk nor restricted to hospitalized patients, is an unmet necessity. Accordingly, the purpose of our study was to investigate short-term outcomes in ED AHF patients included in the Epidemiology of AHF in the Emergency Departments (EAHFE) registry.

## Methods

### Setting and patient selection

Extensive details about the EAHFE registry have been reported elsewhere [2, 3, 21]. Briefly, EAHFE is a prospective multicenter registry which includes patients with AHF attended to Spanish EDs, independently of their final disposition (e.g., admission to a general ward, admission to intensive care unit or discharged home). The current study is a specific secondary analysis of a multipurpose analysis. Diagnosis is initially based on clinical criteria and, whenever it is possible, confirmed by natriuretic peptides or echocardiographic criteria, as recommended by the ESC guidelines [6]. For our analysis, eligible patients were consented and enrolled from 11 Spanish emergency departments participating in the phase EAHFE registry 6 (January–February, 2018) and 7 (January–February, 2019) and met the entry criteria of an ED ECG with QTc measurement available. The sole exclusion was the presence of a ventricular paced rhythms (thus precluding accurate QTc measurement) on the initial ECG. All ECGs were standard 12‐lead resting ECGs (25 mm/s paper speed, 10 mm/mV amplitude, and 250 Hz sampling rate) and were inspected visually for quality and incorrect measurements. Extreme values were visually verified or excluded. The QTc value was provided by the electrocardiograph and it was calculated using the Phillips QT-interval automatic measurement DXL algorithm. The measurement accuracy of the DXL Algorithm has been measured on the ECGs specified by the IEC 60,601–2-51 standard for safety and performance of analyzing electrocardiographs. The QT interval was measured from the beginning of the QRS complex to the end of the T wave, identifying the inflexion point to determine end of T wave defined as the return of voltage to the isoelectric line. *(Philips DXL ECG Algorithm Physician’s Guide).* The QT corrections for heart rates were conducted using the Fridericia's cube root exponential formula [9, 22]. We chose Fridericia’s formula as it is considered to have higher sensitivity than Bazett’s formula in detecting QT prolongation than Bazett’s formula In our study, we used the Fridericia’s correction as it is the most frequently displayed in electrocardiographs used in Spanish ED (while Bazett’s formula is more frequently used in US, Fredericia’s formula is used in may electrocardiographs in Europe). and for centers using Bazett’s formula, Bazett-QTc duration was transformed to Fridericia-QTc duration before statistical analyses.

### Independent variables

We recorded age, sex, and 12 variables corresponding to comorbidities that included hypertension, dyslipidemia, diabetes mellitus, coronary artery disease (CAD), heart valve disease, peripheral artery disease, cerebrovascular disease, chronic kidney disease (defined as creatinine > 2 mg/mL), chronic obstructive pulmonary disease (COPD), dementia, active neoplasia and liver cirrhosis. Baseline status was recorded as 3 variables consisting of functional class according to Barthel index, respiratory class according to New York Health Association (NYHA) and left ventricular ejection fraction (LVEF). Chronic treatment was evaluated by the following medications: diuretics, renin-angiotensin system (RAS) inhibitors, betablockers, mineralocorticosteroid receptor antagonists (MRA) and digoxin), and 6 triggers of decompensation were included; infection, rapid atrial fibrillation (defined by a heart rate > 120 bpm plus need of treatment to control such a rapid rhythm), anemia, dietetic-therapeutic transgression, acute coronary syndrome and hypertensive crisis. Vital signs at ED arrival (systolic blood pressure, heart rate, pulse oximetry), 6 lab results (hemoglobin, creatinine, sodium, potassium, NT-proBNP and troponin I) and ECG findings in addition to QTc duration (atrial fibrillation, left-bundle branch block -LBBB-, left ventricular hypertrophy) were documented.

### Endpoint

We considered all-cause mortality during the following 30 days after ED presentation as the primary outcome to assess the severity of the decompensation. As secondary outcomes, we included the need for hospitalization, in-hospital mortality for hospitalized patients and prolonged hospitalization (> 7 days) if discharged from the hospital. Outcome adjudication was performed at the local level by the principal investigator from each center, without independent external review. For this purpose, local investigators telephonically contacted the patient or their relatives, reviewed the patients’ medical reports and/or consulted the Spanish death registry.

### Statistical analysis

Quantitative variables are expressed as median and interquartile range (IQR), and qualitative variables as the number of patients and percentages. To avoid dichotomizing QTc duration into a few discrete ordered levels and to avoid imposing linearity, we used an RCS function to model the continuous association of QTc duration and primary and secondary outcomes. Five spline knots were placed at the 5, 27.5, 50, 72.5 and 95 centiles of each continuous variable marginal distribution, following the recommendations of Harrel [23]. The magnitude of the effect of each QTc duration unit change on unadjusted outcomes was graphically assessed. Because the continuous QTc duration was modeled with RCS, its unadjusted and adjusted associations were expressed in a dose–response manner for probability or odds ratio (OR) with 95% confidence intervals (CI) for each outcome of interest. To compute an OR for the dose–response plots, we a priori chose arbitrarily the QTc duration of 450 ms as the reference value. Adjustment was performed for patient baseline and decompensation characteristics previously cited. Missing values in quantitative variables were replaced by their median, and missing values in qualitative variables were replaced by their mode. Interaction for 20 independent covariates selected for their influence on outcomes with the relationship between QTc duration and primary outcome was assessed in the adjusted model. For this analysis, we transformed non-dichotomic variables into dichotomous variables following clinical meaningful cut-offs.

As widening of the QRS complex –in the setting of LBBB– leads to QT-interval prolongation without significant alterations to the repolarization duration, we also investigated the potential effect of including patients with LBBB in our study. With this purpose and in addition to investigate interaction of LBBB in the relationship between QTc duration and risk of death at 30 days (primary outcome), we also run 2 sensitivity analyses in the adjusted model for primary outcome, which consisted in: (1) excluding patients with LBBB (sensitivity analysis A); and (2) using the Rautaharju’s formula [24] to correct QT duration in patients with LBBB (sensitivity analysis B). The Rautaharju’s formula has been proposed to be more precise for patients with LBBB and cover the complete heart rate spectrum [25].

All hypothesis testing was two-tailed and *p* values < 0.05, or OR with a 95% CI excluding 1, were considered statistically significant. Data analysis was performed using Statistical Package for Social Sciences version 23.0 (IBM, Armonk, NY, USA) and Stata version 16.1 (Stata Corp, College Station, TX, USA), and some graphs were produced using Microsoft Office Power Point version 2019 (Microsoft Corporate Office, Redmond, Washington, USA).

### Ethics

The EAHFE Registry protocol was approved by a central Ethics Committee at the Hospital Universitario Central de Asturias (Oviedo, Spain) with the reference numbers 49/2010, 69/2011, 166/13, 160/15 and 205/17. Due to the non-interventional design of the registry, Spanish legislation allows central Ethical Committee approval, accompanied by notification to the local Ethical Committees. All participating patients gave informed consent to be included in the registry and to be contacted for follow-up. The present study was carried out in strict compliance with the principles of the Declaration of Helsinki.

## Results

**Fig. 1:**
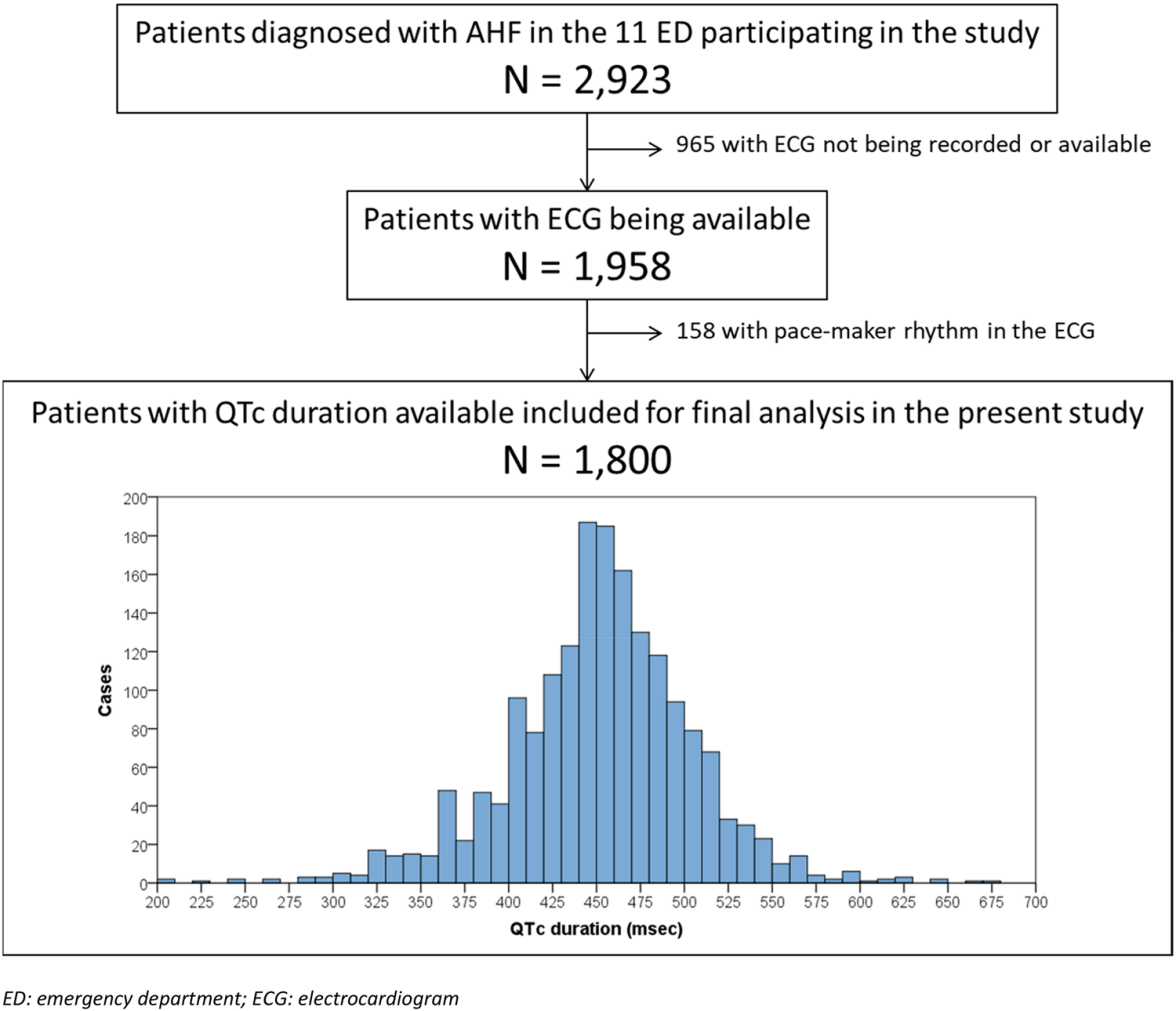
Flow chart for patient inclusion and patient distribution according to the QTc interval duration. *ED* emergency department, *ECG* electrocardiogram

We analyzed 1,800 AHF patients for whom an ECG was performed, with a median QTc duration of 453 ms (IQR = 422–483) (Fig. 1). The median age was 84 years (IQR = 77–89), and 56% were women. Overall, reporting rates were very high (above 95%) for almost all population characteristics. Most common comorbidities were hypertension (81%), dyslipidemia (44%), diabetes mellitus (37%) and chronic kidney disease (30%). Additionally, most patients had some degree of functional dependence (Barthel index < 100 points) at baseline in 66%, and respiratory capacity was affected in 76% (NYHA class II–IV). Although left ventricular ejection fraction was reported in only 67% of patients, it was found to be reduced (defined as < 40%) in 25% of patients. The most frequent chronic treatments were diuretics (73%) and RAS inhibitors (51%), but the infrequent use of other heart failure therapies may suggest that goal directed guideline compliant management may have been underutilized.

Overall, the main triggers of decompensation were infection (43%) and rapid atrial fibrillation (14%). Although not considered the cause of decompensation, atrial fibrillation was found on the ECG of 50% of patients. In terms of laboratory data, 70% of patients had a troponin result above the 99th centile, although this was tested in only 57.3% of patients. Additionally, 37% had a very high level of NT-proBNP (> 5000 pg/mL), although this was only tested in 75.9% of patients. Finally, potassium measurement was obtained in all but 8.2% of patients. While potassium abnormalities may alter QTc, this small value is unlikely to have impacted our results. The remainder of baseline and decompensation characteristics are detailed in Table 1.

**Table 1:** Patient characteristics included in the present study

	Total *N* = 1800 *n* (%)	Missing values *n* (%)
Baseline patient characteristics		
Demographic data		
Age (years) [median (IQR)]	84 (77–89)	1 (0.1)
Female	1004 (55.8)	1 (0.1)
Comorbidities		
Hypertension	1455 (81.5)	14 (0.8)
Dyslipidemia	786 (44.0)	14 (0.8)
Diabetes mellitus	662 (37.1)	14 (0.8)
Chronic kidney disease (creatinine > 2 mg/mL)	533 (29.8)	14 (0.8)
Chronic obstructive pulmonary disease	436 (24.4)	14 (0.8)
Coronary artery disease	422 (23.6)	14 (0.8)
Heart valve disease	406 (22.7)	14 (0.8)
Active neoplasia	278 (15.6)	14 (0.8)
Cerebrovascular disease	231 (12.9)	14 (0.8)
Dementia	206 (11.4)	14 (0.8)
Peripheral artery disease	166 (9.3)	14 (0.8)
Liver cirrhosis	25 (1.4)	14 (0.8)
Baseline status		
Barthel Index (points) [median (IQR)]	90 (70–100)	47 (2.6)
NYHA class		48 (2.7)
I	413 (23.6)	
II	891 (50.9)	
III	427 (24.4)	
IV	21 (1.2)	
Left ventricular ejection fraction (%) [median (IQR)]	57 (50–65)	596 (33.1)
Chronic treatments		
Diuretics	1291 (72.7)	25 (1.4)
Renin-angiotensin system inhibitors	913 (51.5)	28 (1.6)
Betablockers	841 (47.6)	32 (1.8)
Digoxin	192 (10.6)	32 (1.8)
Mineralocorticosteroid receptor antagonists	185 (10.3)	28 (1.6)
Characteristics of decompensation		
Triggers of decompensation		
Infection	766 (43.2)	28 (1.6)
Rapid atrial fibrillation	248 (14.0)	28 (1.6)
Anemia	117 (6.6)	28 (1.6)
Dietetic-therapeutic transgression	90 (5.1)	28 (1.6)
Acute coronary syndrome	77 (4.3)	28 (1.6)
Hypertensive crisis	62 (3.5)	28 (1.6)
Vitals at ED arrival [median (IQR)]		
Systolic blood pressure (mmHg)	140 (122–155)	15 (0.8)
Heart rate (bpm)	87 (73–102)	59 (3.3)
Pulse oximetry (%)	94 (90–97)	26 (1.4)
Analytical findings		
Hemoglobin (g/L) [median (IQR)]	122 (108–135)	12 (0.7)
Creatinine (mg/mL) [median (IQR)]	1.1 (0.9–1.5)	18 (1.0)
Sodium (mmol/L) [median (IQR)]	140 (137–142)	71 (3.9)
Potassium (mmol/L) **[median (IQR)]**	4.4 (4.0–4.8)	148 (8.2)
NT-proBNP (pg/mL) [median (IQR)]	3444 (1659–7433)	433 (24.1)
Raised troponin	724 (70.2)	768 (42.7)
ECG findings		
Atrial fibrillation	903 (50.2)	0
Left-bundle branch block	192 (10.7)	0
Left ventricular hypertrophy	75 (4.2)	0

**Fig. 2:**
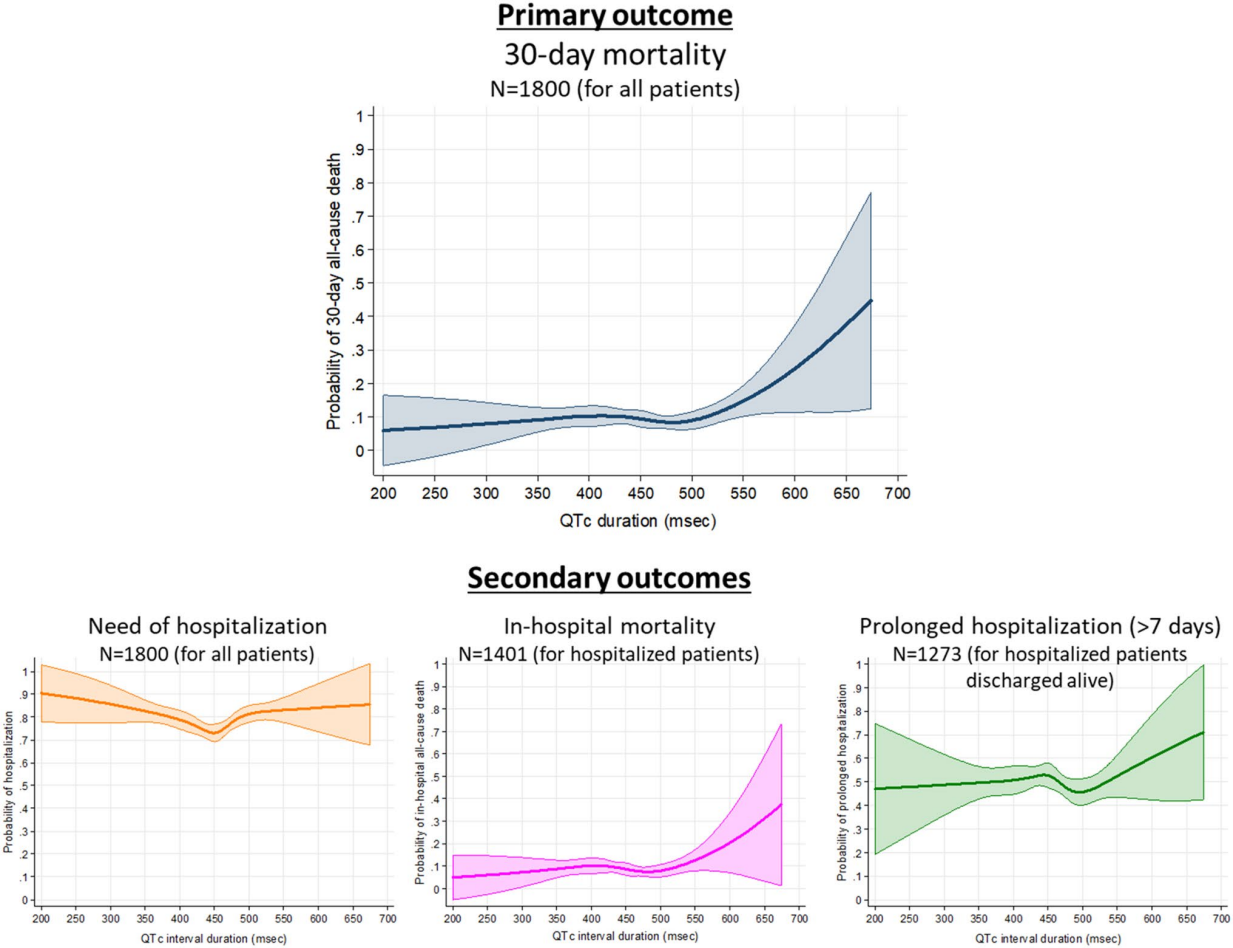
Restricted cubic spline curves showing the observed probability of primary and secondary outcomes according to the duration of the QTc interval duration

The 30-day mortality was observed in 175 patients (9.7%). The curve of 30-day all-cause death demonstrates a flattened aspect for QTc duration below 500 ms, and then shows a mortality increase in parallel with increasing QTc (Fig. 2). In regards to secondary outcomes, need of hospitalization, in-hospital mortality and prolonged hospitalization rates were 77.8% (1401 patients), 9.0% (126 out of the 1401 hospitalized patients) and 50.0% (637 out of the 1275 patients hospitalized and discharged alive), respectively. The curve for in-hospital mortality shows a similar shape as that observed for the relationship for prolonged hospitalization. The curve for probability of hospitalization has a U-shape, with risk increased for both at the extremes of long and short QTc, while the curve for prolonged hospitalization does not show a clear pattern (Fig. 2).

**Fig. 3:**
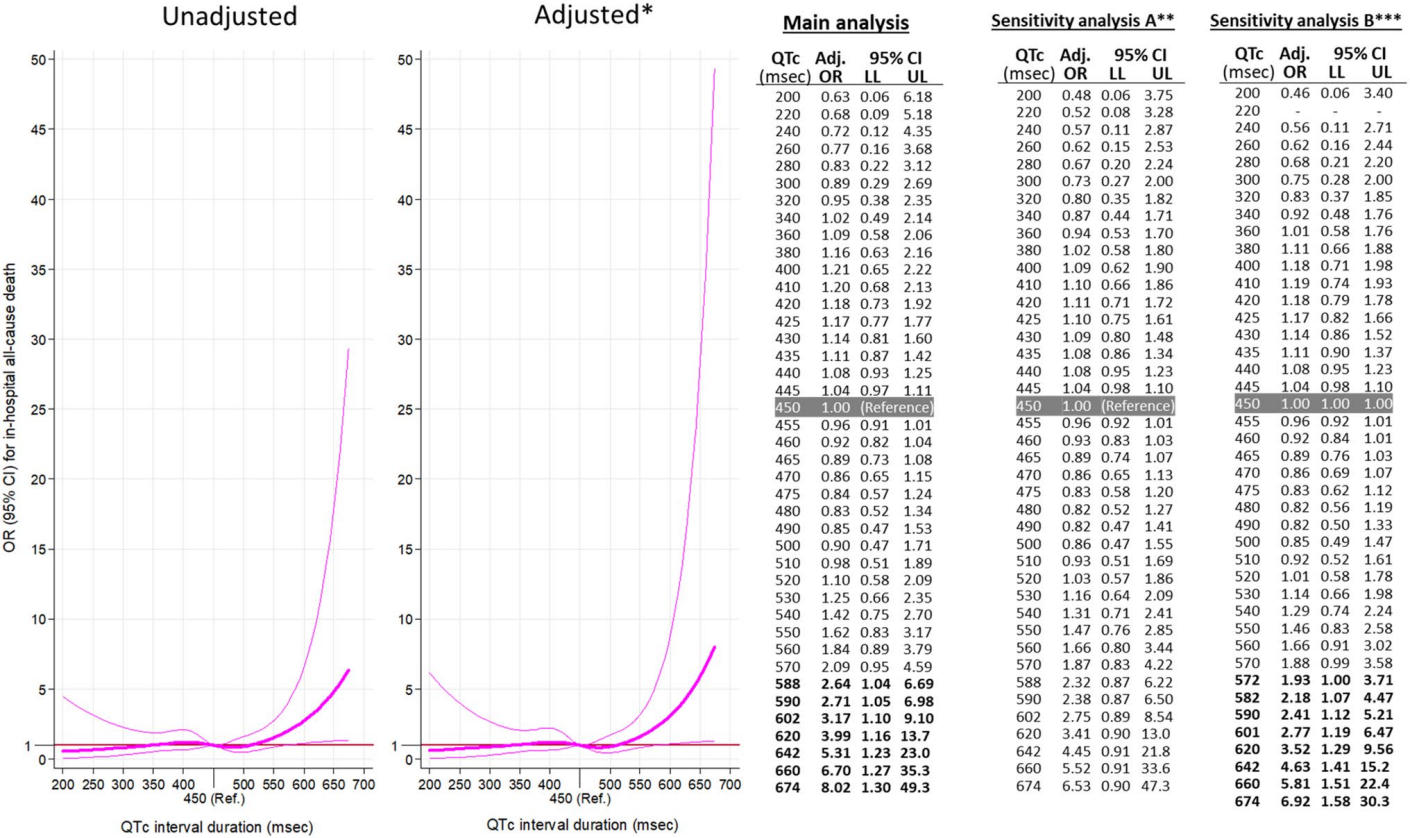
Unadjusted and adjusted representation of the magnitude of the effect of QTc duration on the primary outcome (30-day all-cause mortality) expressed in a dose–response manner as odds ratio (OR) with 95% confidence intervals (CI) taking QTc duration of 450 ms as reference. Tables on the right present some selected OR, with 95% CI (*LL* lower limit, *UL* upper limit), in the adjusted models. Bold numbers in table denote statistical significance (*p* < 0.05)**. **Adjusted by baseline patient characteristics and characteristics of decompensation (Table 1). *Patients with left brunch-bundle block were eliminated in this sensitivity analysis. **QT duration was corrected with the Rautaharju’s formula in patients with left brunch-bundle block

**Fig. 4:**
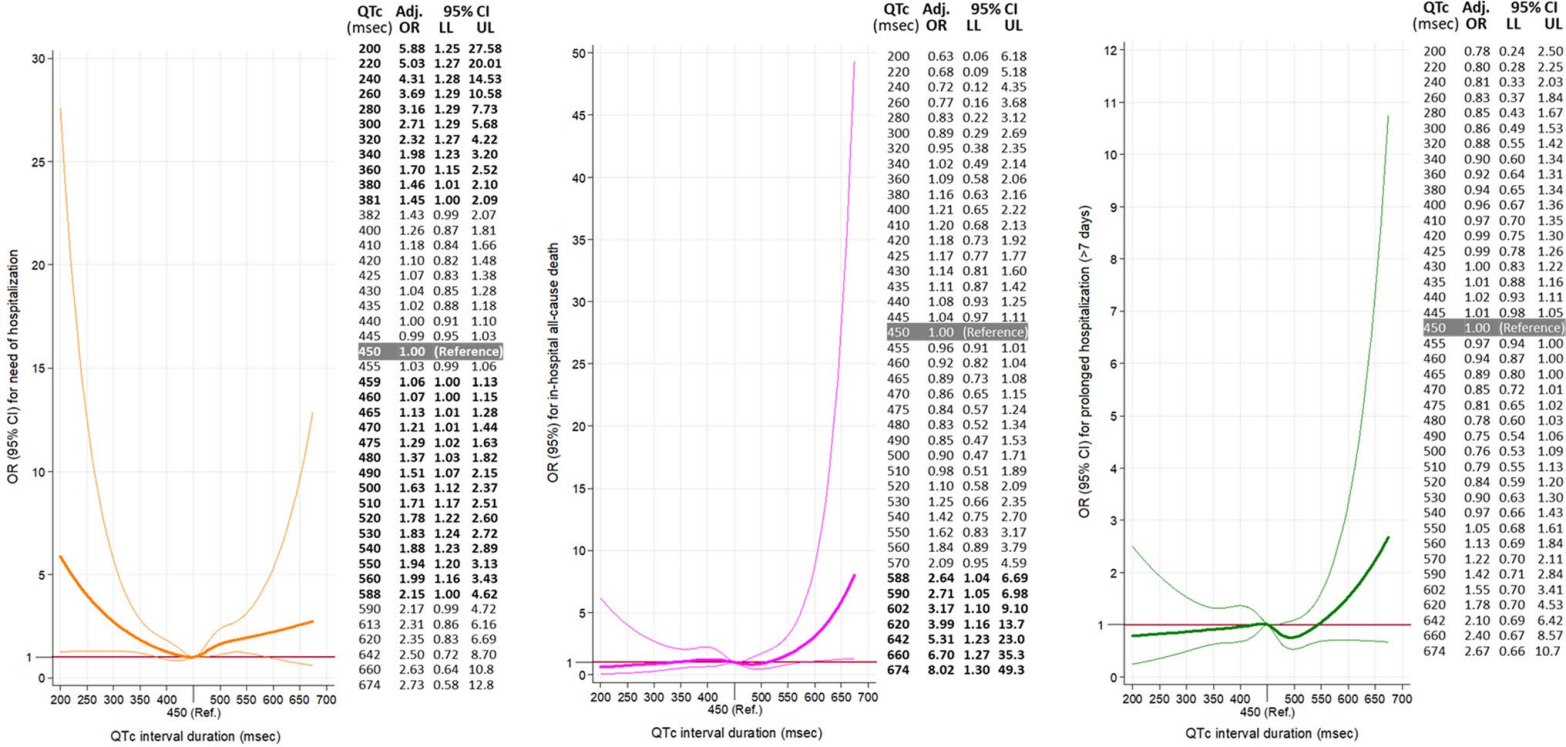
Adjusted* representation of the magnitude of the effect of QTc duration on the secondary outcomes (left: need of hospitalization; middle: in-hospital all-cause mortality; right: prolonged hospitalization) expressed in a dose–response manner expressed as odds ratio (OR) with 95% confidence intervals (CI) taking QTc duration of 450 ms as reference. Tables beside graphs present some selected OR, with 95% CI (*LL* lower limit, *UL* upper limit). Bold numbers in table denote statistical significance (*p* < 0.05). *Adjusted by baseline patient characteristics and characteristics of decompensation (Table 1)

Odds ratio adjusted by patient baseline and decompensation characteristics provides similar curves as in the unadjusted analyses for both, primary (Fig. 3) and secondary outcomes (Supplementary Fig. 1). In the adjusted 30-day mortality model, there is a significant increase in risk from QTc above 561 ms (OR 1.86, 1.00–3.45) and higher, with an OR as high as 10.5 (2.25–49.1) for a QTc of 674 ms (Fig. 3). Sensitivity analyses showed a very similar estimation of risk of death at 30 days associated with QTc duration, with adjusted OR being over 1 from around 580 ms, although due to low number of patients with long QTc duration in both analyses, statistical significance was not present for estimations (Fig. 3). A similar pattern was observed for in-hospital mortality, with QTc above 588 ms having an OR 2.64 (1.04–6.69) and reaching the highest OR of 8.02 (1.30–49.3) for QTc of 674 ms (Fig. 4). Hospitalization risk demonstrated a U-shape characteristic, and was highest at a QTc 200 ms, OR = 5.88 (1.25–27.6) and at QTc = 588 ms, OR = 2.15 (1.00–4.62). After this point, although OR still increased in some extend up to 2.73 for QTc = 674 ms, it does not achieve statistical significance (95% CI 0.57–12.8, Fig. 4). The risk of prolonged hospitalization was not associated with QTc duration (Fig. 4).

**Fig. 5:**
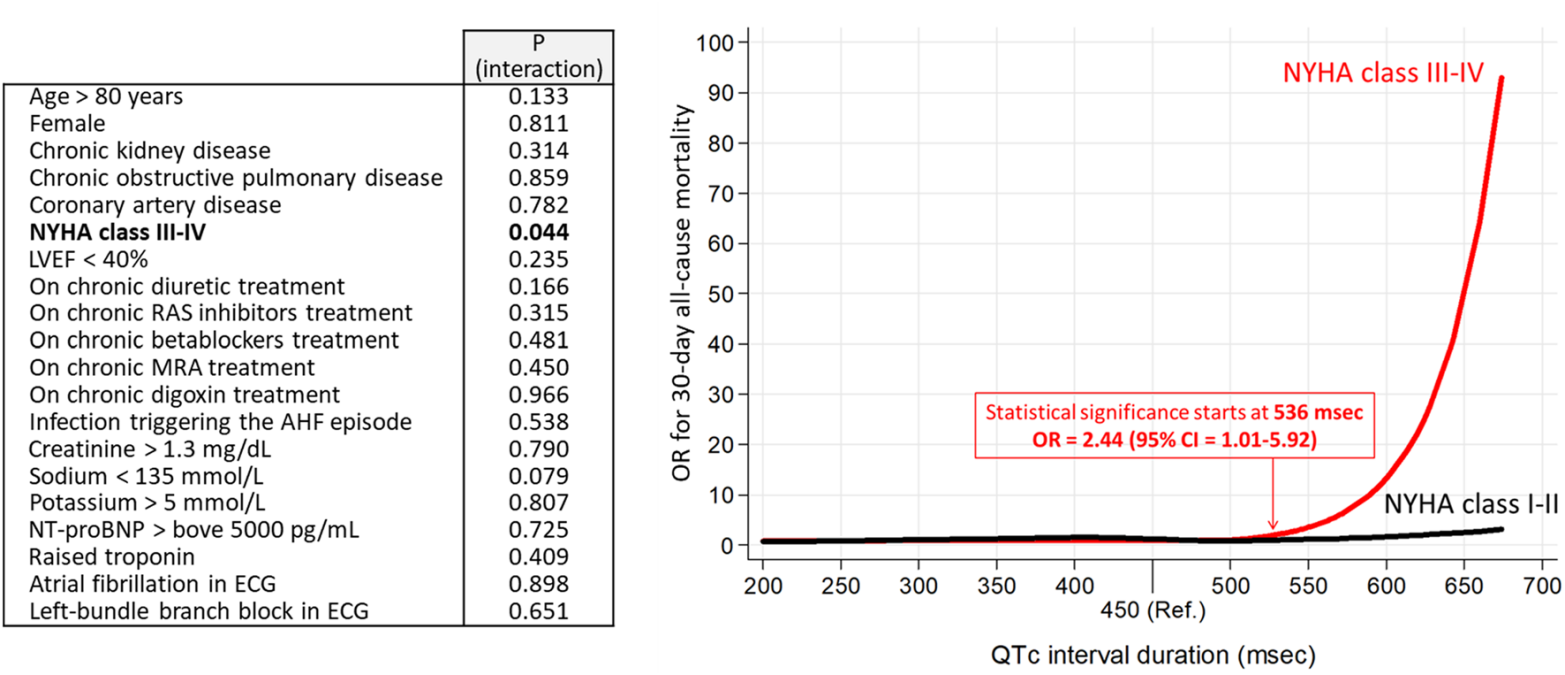
Analysis of interactions in the adjusted model (with imputation) for 20 selected variables in the relationship between QTc interval duration and the primary outcome (30-day all-cause mortality). Bold *p* values denote statistical significance (< 0.05). *NYHA* Ney York Health Association, *LVEF* left ventricular ejection fraction, *RAS* renin-angiotensin system inhibitors, *MRA* mineralocorticosterid receptor antagonists, *OR* odds ratio, *95% CI* 95% confidence interval

Analysis of interaction showed that adjusted OR curves for 30-day mortality was only different depending on the NYHA class of patient, being prolonged QTc a significantly worse risk factor for dying in patient on class III–IV than in patients on class I–II (Fig. 5). For the remaining 19 variables tested, no interaction was found (Fig. 5).

## Discussion

We have found that severity of decompensation in ED AHF, when assessed by the rate of hospitalization, is “U” shaped, with higher rates of admission for QTc below 381 ms, or above 459 ms. Additionally, a prolonged QTc (> 561 ms) is associated with increased risk of 30-day mortality as well as with increased in-hospital mortality risk (> 588 ms), but was not associated with prolonged hospitalization. Notably, all these findings were consistent even after adjusting for age, sex, multiple comorbidities and baseline conditions, as well as for characteristics of decompensation. Remarkably, it is important to note that few studies in AHF patients have been performed in the ED using the first ECG recorded in ED, and that we have used a continuous analysis of QTc duration, which have demonstrated that risk of dying is progressively increased above 560 ms. As QTc interval has not usually been considered in risk models of patients with HF (AHF), we suggest potential of measurement of QTc duration in risk stratification of patients with AHF should be tested in the future.

Our findings are congruent with previous studies demonstrating an association between prolonged QTc interval and increased mortality in chronic HF patients [15, 16]. However, our study was unique because most prior studies evaluated long-term prognosis, while ours examined short-term outcomes in AHF patients. Interestingly, the nadir for mortality in our study was around 480 ms and represents a right-shift as compared to that in chronic HF [15]. This fact agrees with the recent findings of Park et al., who also reported right-shifted specific nadir in the relationship between QTc duration and long-term mortality (about 440–450 ms in males, and about 470 –480 ms in females) [20]. However, we did not find an increase in mortality for shortened QTc, as Park et al. found, where a J-shaped association was reported. IEven for extremely short QTc durations, we found a very low risk of death, although this was not statistically significant probably due to the limited number of cases with those values. This lack of association of shortened QTc with mortality may appear contradictory to the findings in other chronic HF studies. However, it is possible that the negative effects of a short QTc interval manifest slowly in chronic HF patients, and this is the reason why it was only captured by a long-term follow-up as previously reported by Park et al. (mortality at 44 month) but not in ours (in-hospital and 30-day mortality). Remarkably, patients at advanced stages of HF, those with a baseline respiratory NYHA class III–IV, resulted highly and distinctively vulnerable to prolonged QTc (in comparison with patients at NYHA class I-II).

It is well described that hospitalization rates demonstrate great variability in clinical practice, from country to country, and even from ED to ED [26]. They are highly influenced by subjective perceptions and sometimes hospitalization rates may not match the severity of decompensation in individual patients [27]. The same variability can be applied for length of hospitalization, and prolonged hospitalization as an outcome may even be a less precise and more subjective variable. To some effect, this is demonstrated when comparing the length of hospitalization between Europe and the United States [28, 29], where the former has generally longer inpatient stays. However, we believe that hospitalization rates for AHF can be a surrogate for severity, as the majority of severely decompensated AHF patients are hospitalized and stay longer, while less severe are more likely to be discharged without hospital admission and, if hospitalized, will stay for a shorter period. Thus, a unique, and previously unreported, feature of our findings is the U-shaped curve of hospitalization associated with the extremes of both high and low QTc. Because our study was not designed to identify the underlying physiology linking QTc interval and hospitalization rates, we are unable to fully explain the causal relationship between shortened QTc duration and the need for admission. Hypothetically, the association of QTc interval with the need for hospitalization could be an effective of variation in sympathetic and parasympathetic tone, which are altered in HF patients in general, and to a greater extent in stressed AHF patients in particular, with catecholamines being markedly increased [30, 31]. This hypothesis is supported by several experimental studies suggesting a relatively short QTc duration and repolarization time could be a substrate for multiple-circuit reentry excitation, which would induce atrial/ventricular arrhythmia [32, 33], further worsening the clinical presentation and thus the association with hospitalization. Further studies will need to evaluate the potential for a low QTc admission cut point requirement.

Last, but not least, our study underlines the importance of a more flexible, dynamic exploration of the relationship between a linear variable, like QTc duration, with an outcome. Certainly, the selection of a specific cut point can ignore important relationships between pathology, physiology, and prognosis However, it is particularly challenging for the clinician to accurately discern slight variations in QTc and use of it in the context of subtle clinical presentations to predict outcomes. Hence, there is an opportunity for artificial intelligence applications to outperform clinicians [34, 35]. Some have demonstrated that machine earning pattern recognition may perform better than physicians using dichotomous cut points. This same technology may offer solutions for the QTc in the near future.

### Limitations

There are some limitations that should be considered. First, as in every observational study, causal relationships cannot be inferred. For example, some data were not recorded in many patients (troponin and natriuretic peptides were lacking in 42% and 24% of cases). Therefore, the results of the current analysis are limited by the retrospective design, as should be considered as hypothesis generating. Second, adjudication of outcomes was made locally, by principal investigators of each center without external overview. However, we used easily identifiable and unequivocal outcomes to overcome the need for external adjudication. Third, although we recorded chronic treatment with digoxin, beta-blockers and the presence of infection or acute coronary syndrome as triggers of the AHF episode (and they were included as covariates in the adjusted model), data on other well-known drugs (such as amiodarone) and medical conditions (such as fever) prolonging QTc were not recorded. Fourth, we did not investigate if diagnostic or therapeutic approaches intending to correct QTc duration were made during hospitalization in patients with abnormal QTc. Therefore, the potential of such correction to improve outcomes is not assessed by the present study. Fifth, Spain has a nationwide universal public health care system, and external validation of our results might be needed to confirm their generalizability. Although performed within a single country, organization of care to patients with HF is not uniform in all Spanish territories [36, 37], and strategies of ambulatory follow-up of patients, specially of those at highest risk of complications, can affect outcomes. Sixth, because this study was performed in a “real world” registry environment, with likely lower rates of guideline directed chronic HF therapy, how the unique finding of a U-shaped curve predicting hospitalization could be impacted by greater rates of chronic HF guideline compliant treatment is unclear. Seventh, our study included a high percentage of elderly AHF patients (as the EAHFE Registry includes all patients diagnosed with AHF in the ED with no age limit, and the only exclusion criteria is that AHF is triggered by an ST-elevation myocardial infarction). Accordingly, they predominantly have preserved ejection fraction, as well as frailty and dependence are frequent in very older population, being both conditions strongly related to mortality [38]. Therefore, application of our results in younger cohort of patients should be taken with caution.

## Conclusions

In patients with AHF, initial measurement of QTc duration in ED provides independent information of short-term outcomes: prolonged QTc duration is associated with mortality while both, shortened and prolonged duration are associated with need of hospitalization.

### Supplementary Information

Below is the link to the electronic supplementary material.

Supplementary file1 (DOCX 287 KB)

## Data Availability

The authors confirm that the data supporting the findings of this study are available within the article and its supplementary materials.
