# Supplementary material for: QT interval and short-term outcome in acute heart failure
Source: Clin Res Cardiol. 2023 Apr 1;112(12):1754–65. doi: 10.1007/s00392-023-02173-9 (PMC10698082; doi:10.1007/s00392-023-02173-9)
Supplement: Supplementary file 1 — Supplementary file1 (DOCX 287 KB) [file 392_2023_2173_MOESM1_ESM.docx]

**Supplemental Figure 1:** Adjusted* and unadjusted representation of the magnitude of the effect of QTc duration on the secondary outcomes (left: need of hospitalization; middle: in-hospital all-cause mortality; right: prolonged hospitalization) expressed in a dose-response manner expressed as odds ratio (OR) with 95% confidence intervals (CI) taking QTc duration of 450 msec as reference.


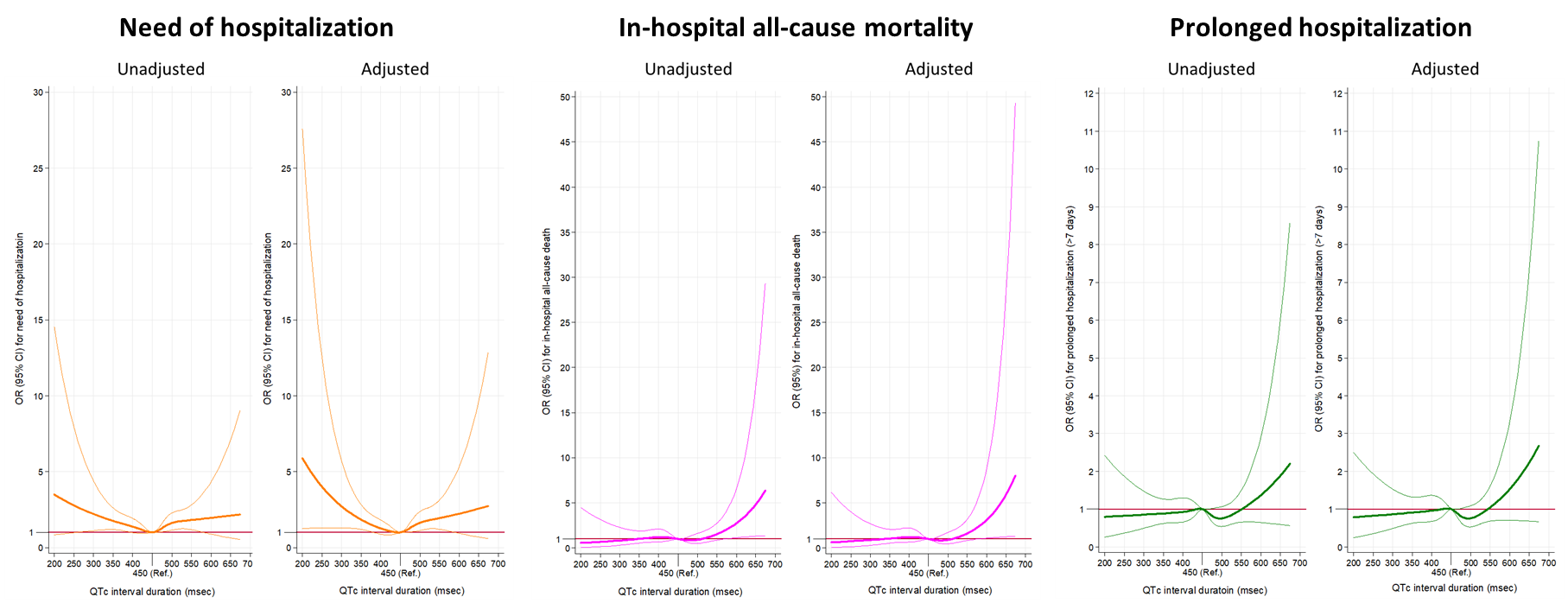


** Adjusted by baseline patient characteristics and characteristics of decompensation (Table 1)*
